# A study of dopant incorporation in Te-doped GaAsSb nanowires using a combination of XPS/UPS, and C-AFM/SKPM

**DOI:** 10.1038/s41598-021-87825-4

**Published:** 2021-04-15

**Authors:** Priyanka Ramaswamy, Shisir Devkota, Rabin Pokharel, Surya Nalamati, Fred Stevie, Keith Jones, Lew Reynolds, Shanthi Iyer

**Affiliations:** 1 Department of Electrical and Computer Engineering, North Carolina A&T State University, Greensboro, NC 27401 USA; 2 Nanoengineering, Joint School of Nanoscience and Nanoengineering, North Carolina A&T State University, Greensboro, NC 27401 USA; 3 Analytical Instrumentation Facility, North Carolina State University, Raleigh, NC 27695 USA; 4 Asylum Research, an Oxford Instruments Company, 6310 Hollister Ave., Santa Barbara, CA 93117 USA; 5 Department of Materials Science and Engineering, North Carolina State University, Raleigh, NC 27695 USA

**Keywords:** Techniques and instrumentation, Nanoscale materials, Electronic properties and materials, Nanowires

## Abstract

We report the first study on doping assessment in Te-doped GaAsSb nanowires (NWs) with variation in Gallium Telluride (GaTe) cell temperature, using X-ray photoelectron spectroscopy (XPS), ultraviolet photoelectron spectroscopy (UPS), conductive-atomic force microscopy (C-AFM), and scanning Kelvin probe microscopy (SKPM). The NWs were grown using Ga-assisted molecular beam epitaxy with a GaTe captive source as the dopant cell. Te-incorporation in the NWs was associated with a positive shift in the binding energy of the 3d shells of the core constituent elements in doped NWs in the XPS spectra, a lowering of the work function in doped NWs relative to undoped ones from UPS spectra, a significantly higher photoresponse in C-AFM and an increase in surface potential of doped NWs observed in SKPM relative to undoped ones. The carrier concentration of Te-doped GaAsSb NWs determined from UPS spectra are found to be consistent with the values obtained from simulated I–V characteristics. Thus, these surface analytical tools, XPS/UPS and C-AFM/SKPM, that do not require any sample preparation are found to be powerful characterization techniques to analyze the dopant incorporation and carrier density in homogeneously doped NWs.

## Introduction

In recent years, III–V semiconductor nanowires (NWs) have attracted significant attention due to their one-dimensional architecture, quantum confinement effects, and a higher tolerance for strain mismatch that allow greater freedom in band gap engineering of material systems in a variety of different nanowire architectures to meet the demands of next-generation optoelectronic devices^1, 2^. To realize advanced devices in a NW configuration successfully, dopant incorporation with abrupt interfaces in a well-controlled manner is essential and enables one to modulate work functions. Unfortunately, the thin film studies on dopant incorporation and carrier concentration cannot be directly translated to NWs due to the dopant’s influence on the growth kinetics and different growth mechanisms along with the axial and radial directions^3^. Also, the commonly used measurement techniques in thin films for the determination of carrier concentration, namely Hall effect, field-effect, capacitance–voltage, and thermoelectric measurements, require highly sophisticated lithography steps^4–10^. Besides, in the case of nanowire field-effect transistors (FET), the uncertainty of the gate capacitance and contact resistance can further limit the accuracy of carrier mobility and concentration values^11, 12^.

In recent years, several characterization methods have evolved for the assessment of dopants in NWs. These are secondary ion mass spectrometry (SIMS)^5, 13^, off-axis electron holography (EH)^14–16^, and atom probe tomography (APT)^17–20^. However, SIMS requires a standard of known dopant concentration and is destructive, as is APT. In the case of the EH technique, sample preparation is complex, and it requires additional information about the NW thickness and homogeneity. Goktas et al.^5^ performed a doping assessment of Beryllium (Be) and Te-doped GaAs NWs using various characterization techniques, namely, the Hall effect, Raman, photoluminescence (PL), and SIMS. For Te-doped NWs, Raman and SIMS indicated carrier concentration on the order of 10^17^ cm^−3^, which was at least one order of magnitude lower than the expected value. It was also found that the carrier concentration of Te-doped NWs could not be determined from PL and the Hall effect due to the lower Te dopant concentration in the NWs. Suomalainen et al.^6^ studied qualitative analysis of Te dopant incorporation in self-assisted GaAs NWs using Raman spectroscopy. Orrù et al.^21^ determined a high carrier concentration of 10^20^ cm^−3^ in Te-doped GaAs NWs using a combination of FET transport measurements, KPFM (Kelvin probe force microscopy), and PL. Hakkarainen et al.^16^ showed a Te concentration of 4 × 10^18^ cm^−3^ in Te-doped GaAs NWs by APT using laser pulses.

Other surface analytical methods, namely X-ray photoelectron spectroscopy/ultraviolet photoelectron spectroscopy (XPS/UPS), in conjunction with conductive-atomic force microscopy/scanning Kelvin probe microscopy (C-AFM/SKPM), provide a set of excellent characterization methods for doping assessment, as they do not require any sample preparation. XPS has been more commonly used to identify the elemental compositions of NWs^22–25^. Park et al.^26^ determined the composition of InAsP NWs, and Timm et al.^27^ investigated the interface composition (NW/thin film) of InAs/Al_2_O_3_ and InAs/HfO_2_. UPS has been used in NWs of Cobalt-doped molybdenum carbide to study the effect of doping^28^ and Ge-doped ZnO^29^ to determine the work function. There has been little work reported on the XPS/UPS evaluation of III-V NWs. In contrast, AFM and SKPM (also known as Kelvin probe force microscopy (KPFM)) are increasingly being used to examine the electrical/photoelectrical characteristics^30–33^ and doping type/profile in III-V NWs^34–41^.

In this work, we investigate the effect of different GaTe cell temperatures on Te dopant incorporation and electronic properties of MBE grown Te- doped GaAsSb NWs using several surface analytical characterization techniques tools, namely XPS/UPS and SKPM along with C-AFM. The extensive research interest in GaAsSb NWs stems from its narrow band gap region covering the important optical telecommunication wavelengths of 1.3 µm and 1.55 µm^42^. Statistical analyses of all four measurement techniques have provided evidence for confirmation of Te incorporation in these GaAsSb NWs. The values of electron density from UPS concur very well with the values of electron density determined from C-AFM. Hence, these surface analytical tools without contact fabrication are found to be a very powerful tool for direct measurement of the dopant incorporation and doping levels in NWs.

## Results and discussion

We discuss four MBE grown samples, intrinsic and Te-doped GaAsSb NWs of GaTe cell temperatures of 500 °C, 550 °C, and 570 °C, which will be referred to as R, N-500, N-550, and N-570 samples, hereafter, respectively. The corresponding diameters are 136 ± 6 nm, 140 ± 5 nm, 152 ± 7 nm, and 157 ± 5 nm, respectively, as measured using field emission scanning electron microscopy (FESEM)^43^. These are shown in supplemental information Figure S1(a–h), with the R sample serving as a reference NW sample. In the following sections, evaluation of the electron concentration from the ensemble and single nanowires (SNW) is described consecutively using a combination of contactless/non-destructive techniques (no deposition of contacts), namely, XPS, UPS, C-AFM, and SKPM.

### X-ray photoelectron spectroscopy (XPS)

First, we have carried out the compositional analysis of samples R, N-500, N-550, and N-570 using the 3d peaks of Ga, As, and Sb elements in XPS. The XPS survey spectra of the elements present in these four samples are shown in Figure S2 of the supplementary information. The typical resolution of the instrument is ~ 0.1 eV. The absence of C 1s and O 1s photoelectron spectral peaks in the survey spectra indicated minimal atmospheric contamination.

**Figure 1:**
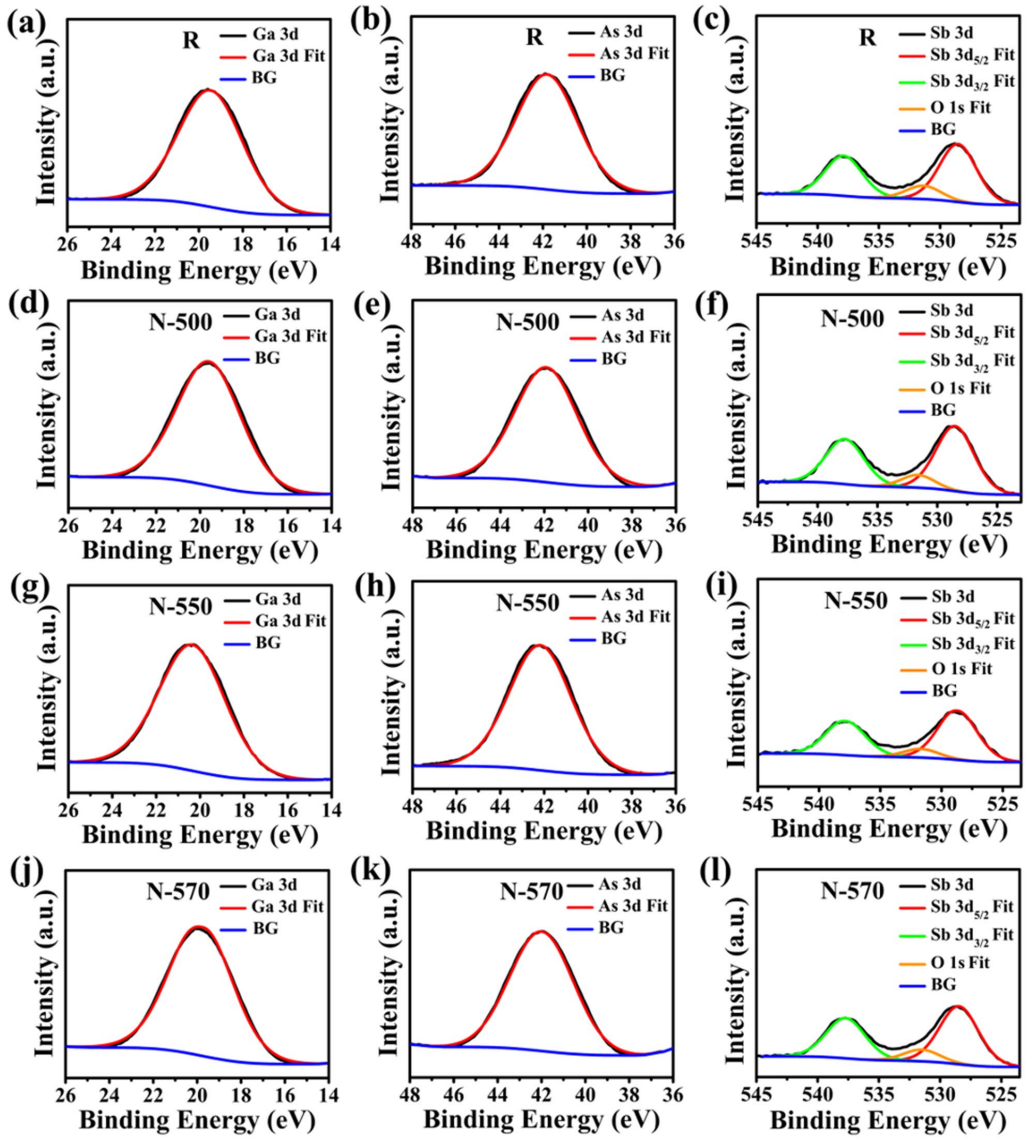
XPS spectra for (**a**,**d**,**g**,**j**) Ga 3d, (**b**,**e**,**h**,**k**) As 3d, (**c**,**f**,**i**,**l**) Sb 3d core levels of (1st row) R, (2nd row) N-500, (3rd row) N-550, and (4th row) N-570 samples, respectively.

**Figure 2:**
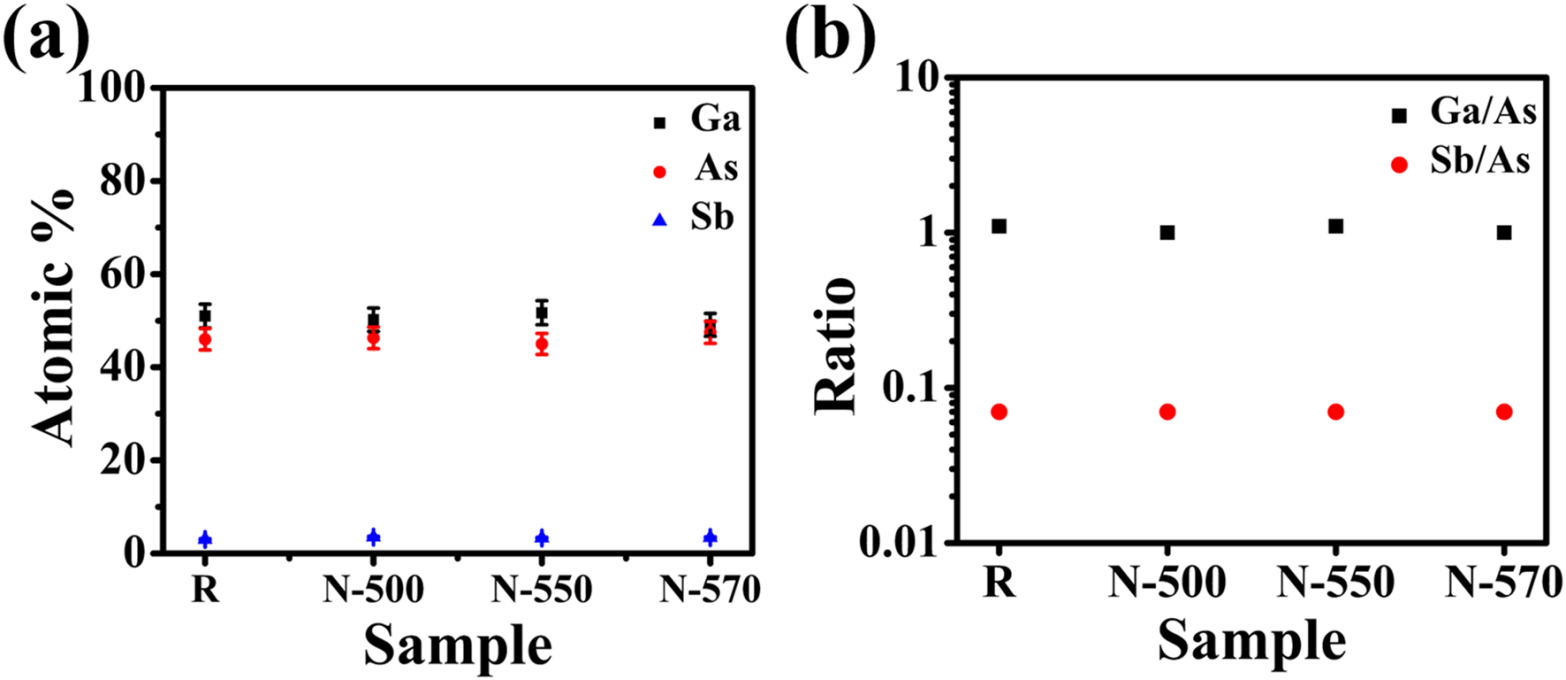
(**a**) Atomic % of Ga, As, and Sb in R, N-500, N-550, and N-570 samples and (**b**) ratio of Ga/As and Sb/As in R, N-500, N-550, and N-570 samples.

Figure 1 shows the core-level XPS spectra, while Fig. 2a summarizes the extracted value of the Ga, As and Sb atomic concentrations in % for R, N-500, N-550, and N-570 samples, and Fig. 2b depicts the ratio of Ga/As and Sb/As in those samples. A Gaussian–Lorentzian (G-L) product function is used for peak fitting of the Sb 3d with the constraint on the area ratio of the Sb 3d doublet (Sb 3d_5/2_:Sb 3d_3/2_) to be 3:2 ratio and a doublet peak separation of 9.4 eV according to the degeneracy of the spin state due to the overlap of the binding energy peaks of O 1s and Sb 3d^44, 45^. The atomic concentration (%) of each element is then calculated as (100 × normalized area of a peak)/(sum of normalized areas of all the peaks).

The atomic ratios (Fig. 2b) of Ga/As for R, N-500, N-550, and N-570 samples are found to be 1.1, 1.0, 1.1, and 1.0, respectively, while for Sb/As, they are 0.07 for all the four samples. These data represent the surface composition and are in excellent agreement with our earlier reports^43, 46^ of concentration determined from the EDS compositional scans of GaAs_0.93_Sb_0.07_ NWs in a transmission electron microscope (TEM).

The peaks observed at 19.5 eV and 41.8 eV in the R sample that are assigned to the Ga 3d and As 3d in GaAs^47–49^ (Fig. 1a,b), respectively, are shifted towards higher binding energy by 0.2 eV and 0.1 eV, in the N-500 sample (Fig. 1d,e), 0.9 eV and 0.4 eV, in the N-550 sample (Fig. 1g,h), and 0.4 eV and 0.2 eV, in the N-570 sample (Fig. 1j,k). It is noted that though the values of binding energy shifts of the N-500 sample are comparable to the resolution of the instrument of 0.1 eV, the observed shifts were obtained at six different spots and the shifts were consistent and reproducible. Also, the NW diameter exhibited a slight increase with respect to the R sample. All of these observations suggest the onset of Te incorporation in the NWs. The peaks observed in R and N samples at 528.6 eV and 538 eV (Fig. 1c,f,i,l) are associated with the doublet Sb 3d_5/2_ and Sb 3d_3/2_, respectively^50^, attributed to Sb–Sb bond and 531.8 eV corresponds to O 1s^44^. From the XPS core-level spectra, the concentration of O 1s is found to be < 1% in the intrinsic and doped GaAsSb samples. The large FWHM broadening of all the peaks observed in all the samples is attributed to the one-dimensional configuration of NWs, leading to enhanced contribution from surface roughness, electron shadowing effect, and shadowing effect of NWs^51^ in comparison to the thin film counterpart. Further, the absence of Te 3d peak^52, 53^ in the N samples suggests that the concentration of Te is less than 10^19^ cm^−3^. The XPS lower detection limit of Te is in the mid-10^19^ cm^−3^, it is further difficult to detect the dopant concentration in the range of detection limit in a ternary alloy. Hence, XPS surface analysis only provides a qualitative comparison of the dopant level. The shifts of Ga and As associated 3d peaks toward higher binding energies indicate increased free-electron density-induced shifts of the core elements, whereas the Sb 3d peak did not exhibit any shift. In GaAsSb, Ga can form bonds with As, Sb, as well as with Te in Te-doped GaAsSb. These peak shifts reflect corresponding shifts in the Fermi level higher in the band diagram, as the binding energies are measured relative to the Fermi level. Therefore, the N-550 sample is found to be heavily doped as compared to other samples N-500 and N-570.

### Ultraviolet photoelectron spectroscopy (UPS)

**Figure 3:**
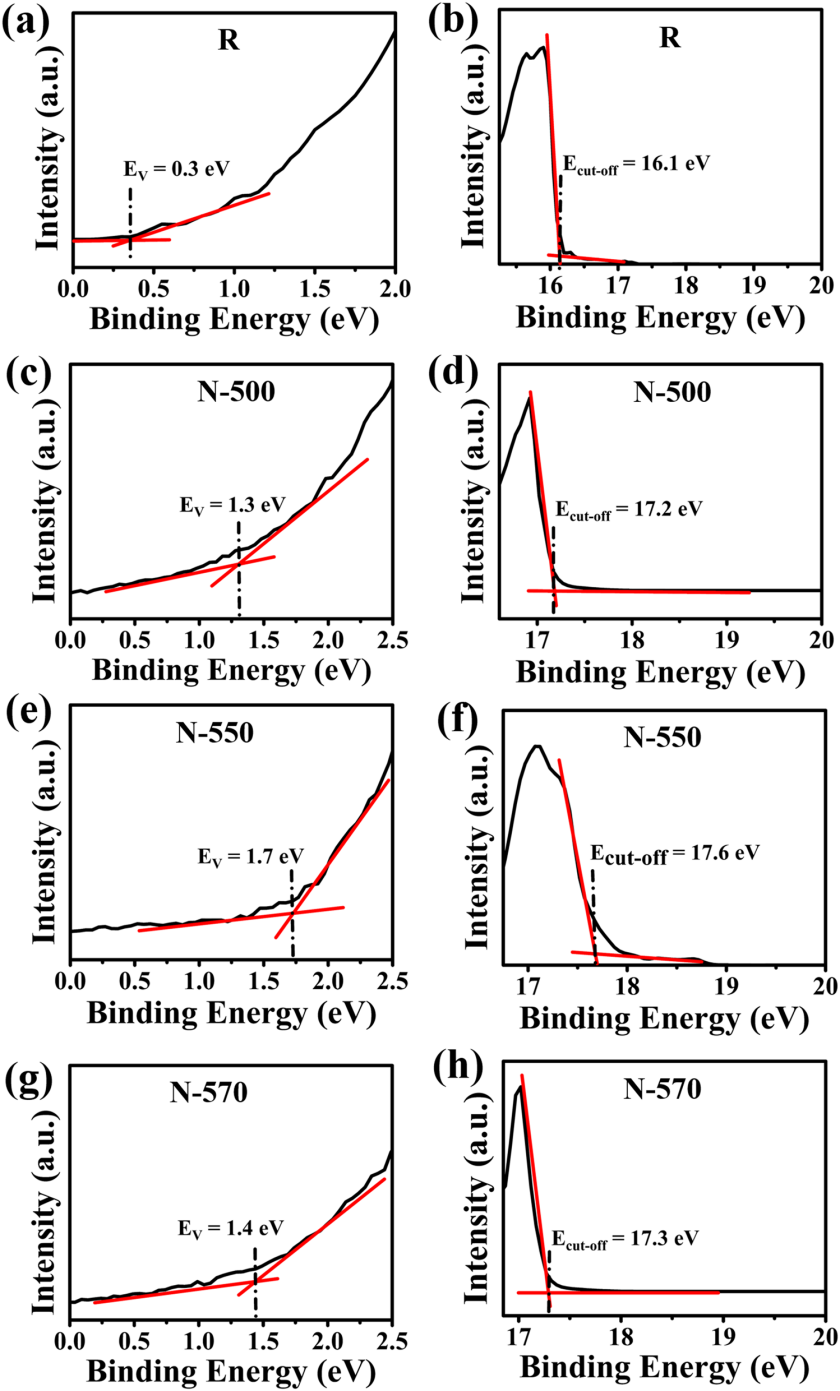
(**a**,**c**,**e**,**g**) Low-BE slope and (**b**,**d**,**f**,**h**) high-BE slope of the UPS spectra of R, N-500, N-550, and N-570 samples, respectively.

Next, electron concentration in the N samples was evaluated using UPS, which can provide a direct measurement of the work function and valence band energy. The work function is extracted from the difference between the energy of the UV photons and the secondary electron cutoff (high-binding energy (BE) cutoff). Figure 3a–h show low and high BE slopes of He I spectra of R, N-500, N-550, and N-570 samples, and also entire UPS spectra of the corresponding samples are shown and summarized in supplementary Figure S3. The intersection of the slope of the high BE with the extrapolated slope of the background electron intensity yielded work function values of 5.1 eV, 4 eV, 3.6 eV, and 3.9 eV for R, N-500, N-550, and N-570 samples, respectively, while similar intersection point at lower BE enabled the determination of the corresponding values of the valence band maximum (VBM) location to be at 0.3 eV, 1.3 eV, 1.7 eV, and 1.4 eV below the Fermi level^54^. The significant difference between the work function of R and N-550 is evident, with N-550 being heavily doped. Using the Fermi–Dirac integral (F_1/2_(${\upeta }_{\mathrm{F}}$))^55, 56^ values of 2.2, 64.0, 13.0 derived from the UPS spectra, an electron concentration of 9.7 × 10^17^ cm^−3^, 2.8 × 10^19^ cm^−3^ and 5.7 × 10^18^ cm^−3^ for N-500, N-550, and N-570, respectively, was calculated using Eq. (1).

1 $${n}_{0}= \frac{2}{\sqrt{\pi }} {N}_{C}{F}_{1/2}\left({\eta }_{F}\right)$$

where n_0_ is the electron concentration, N_C_ is the effective density of states in the conduction band, and F_1/2_(${\upeta }_{\mathrm{F}}$) is the Fermi–Dirac integral. The parameters used are shown in the supplemental information (equations S1 and S2). This value is in excellent agreement with the value obtained from COMSOLand Padovani-Stratton fit^57^, which will be discussed in the following section (Fig. 6e).

### Conductive-atomic force microscopy (C-AFM) and scanning Kelvin probe microscopy (SKPM)

**Figure 4:**
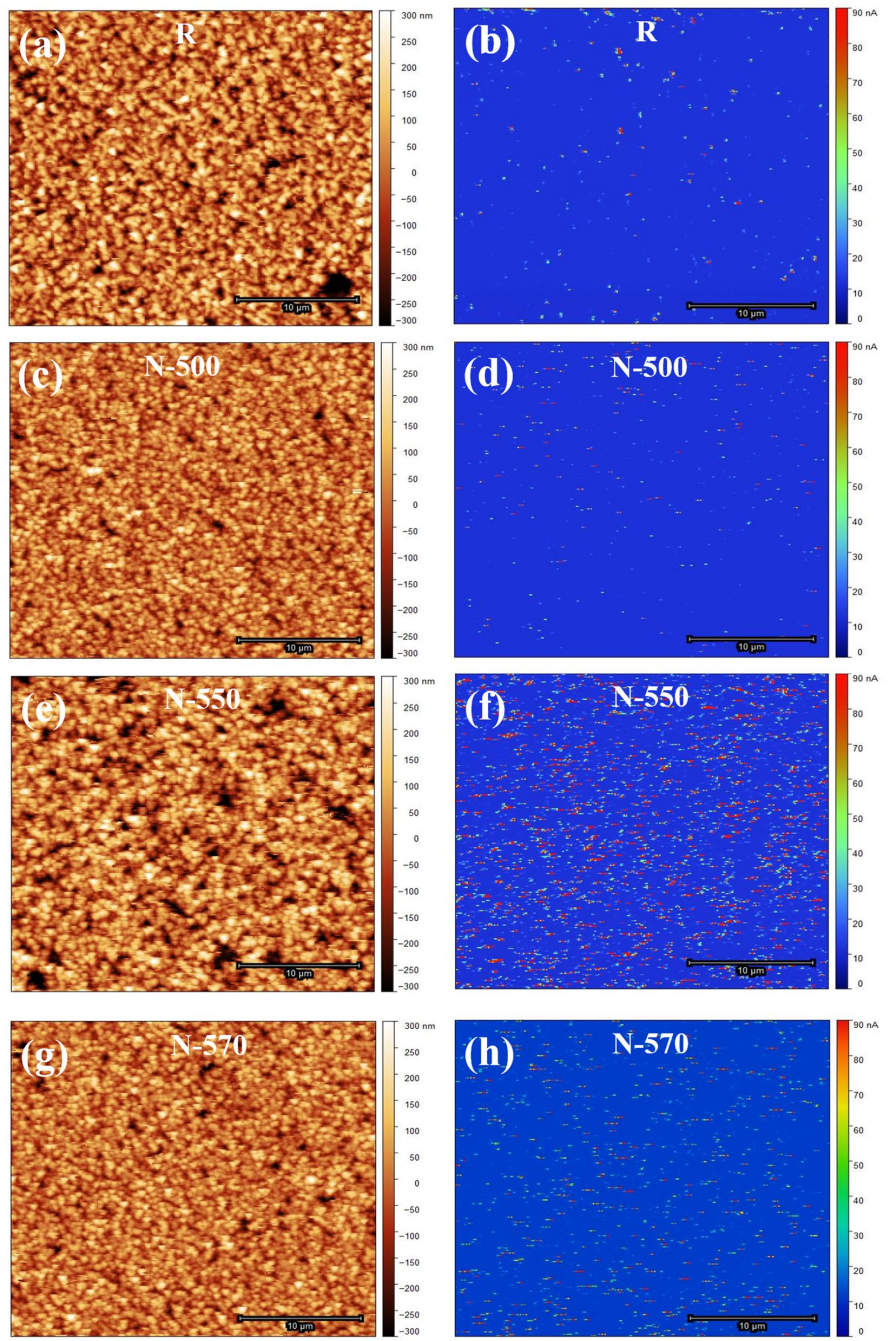
(**a**,**c**,**e**,**g**) Topography and (**b**,**d**,**f**,**h**) corresponding current map of R, N-500, N-550, and N-570 sample, respectively, using C-AFM.

**Figure 5:**
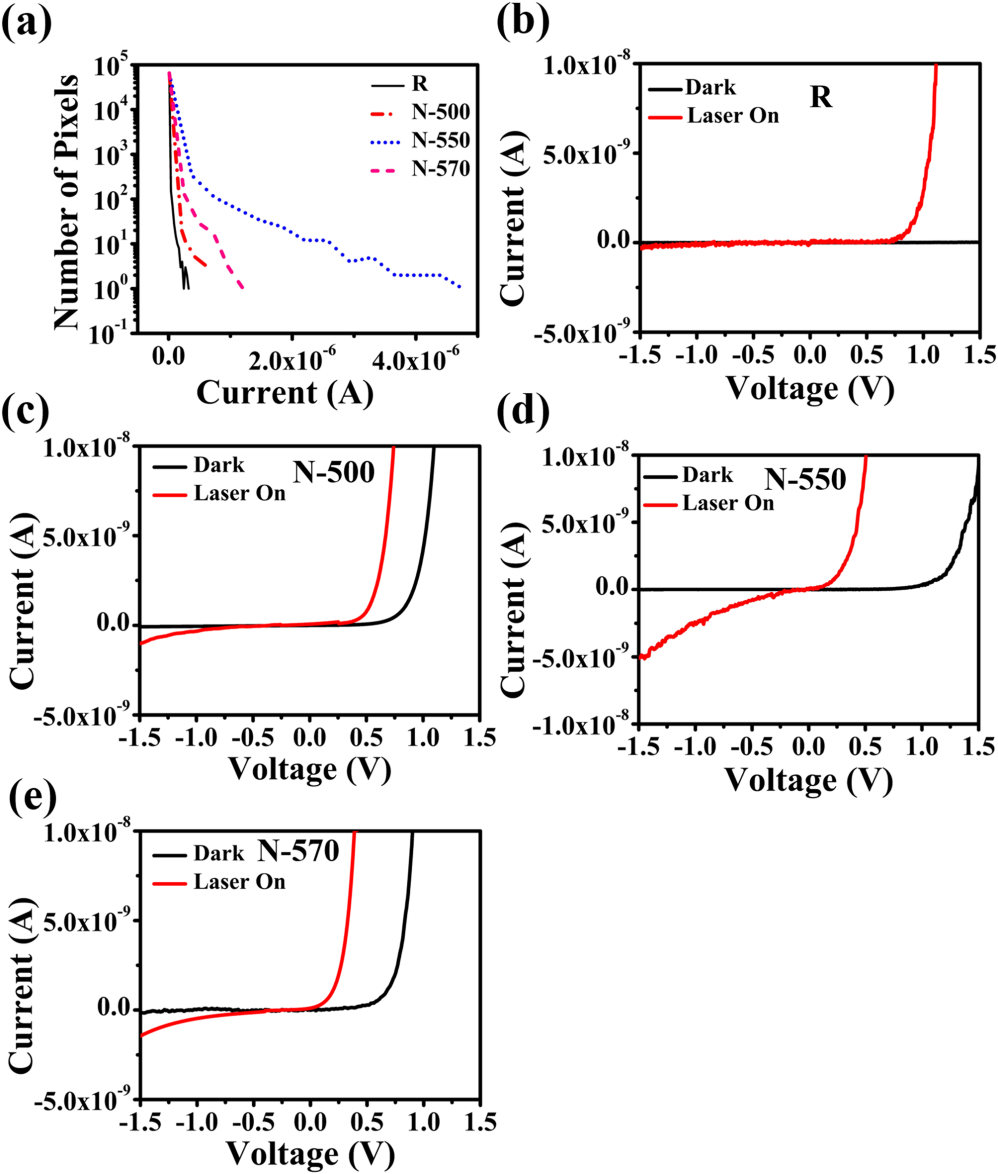
(**a**) Histogram of the current map of samples R, N-500, N-550, and N-570 samples, I–V characteristic of (**b**) R, (**c**) N-500, (**d**) N-550, and (**e**) N-570 samples using C-AFM.

Next, the carrier concentration of the N samples was also determined from the I–V characteristics using C-AFM**.** Figure 4a,b represent the topography and the corresponding current map of an array of vertically aligned R NWs, while Fig. 4c–h represent those of N NWs. Figure 5a shows the current map histogram (number of pixels vs current) of R, N-500, N-550, and N-570 samples, plotted with points. It is found that the background of the four samples is the same; hence, the increase in current is from the nanowires themselves. The histogram indicates both the dramatic increase in current ‘hot spots’ and the significant increase in the average value of the current at those ‘hot spots’ in N samples compared to the R sample, thereby confirming the incorporation of Te in the NWs. From the current mapping, it is evident that the N-550 sample is more conductive. This is also corroborated from the higher photoresponse of 5 nA observed in SNW of N-550 sample (Fig. 5d) compared to 248 pA in SNW of R sample (Fig. 5b).

**Figure 6:**
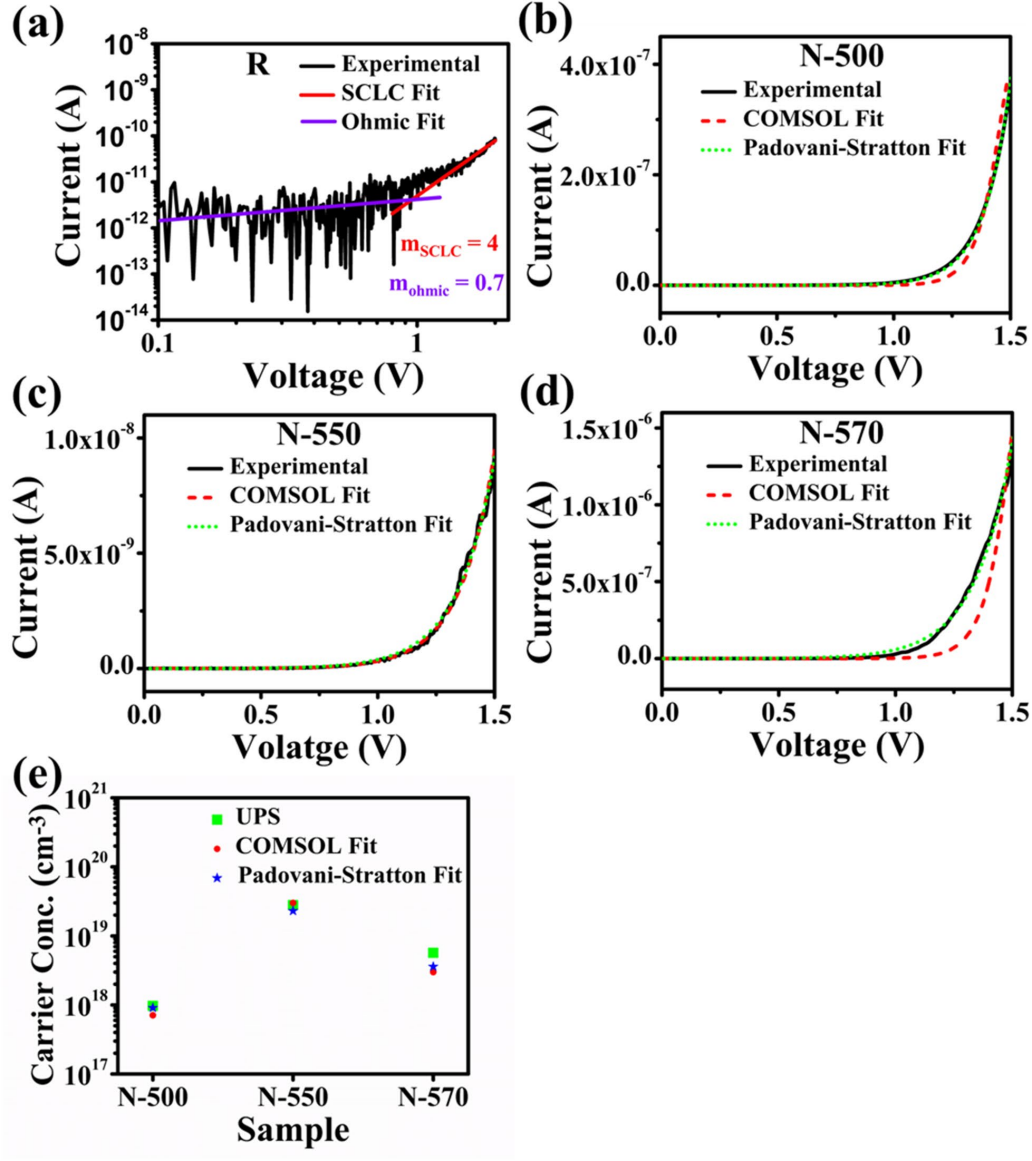
(**a**) Log–log I–V characteristic of R sample exhibiting linear and power dependence, (**b**–**d**) experimental I–V dark, simulated COMSOL fit, and Schottky diode fitting of dark I–V characteristics of (**b**) N-500, (**c**) N-550, and (**d**) N-570 samples, and (**e**) carrier concentration of N-500, N-550, and N-570 samples extracted from UPS, COMSOL fit, and Padovani-Stratton fit.

Figure 6a shows that the I–V curve under forward bias follows a power-law relationship I ∝ V^m^, with m = 4, thereby confirming the trap-associated space charge limited current mechanism (SCLC) exhibited by the R sample^58^. The finite element method (FEM) numerical solving technique was used to model nanowires with Schottky contact on one side and rectifying contact on the other side, using COMSOL Multiphysics simulation software for N sample. Figure 6b–d show the best fit of the simulated I–V characteristics of the nanowires to the experimental data yielding electron mobility values of 1400 cm^2^V^−1^ s^−1^, 1235 cm^2^V^−1^ s^−1^, and 1200 cm^2^V^−1^ s^−1^ for the N-500, N-550, and N-570 samples, respectively, hole mobility of 70 cm^2^V^−1^ s^−1^, with an estimated carrier concentration of 7.1 × 10^17^ cm^−3^, 3.0 × 10^19^ cm^−3^, and 3.0 × 10^18^ cm^−3^, respectively, with an estimated error bar of 5%. It is to be noted that the highest carrier concentration was achieved for GaTe cell temperature of 550 °C. The decrease in carrier concentration at 570 °C is consistent with reduced conductivity observed in the current map of C-AFM and higher dark current along with the decline in photoresponse in the corresponding I–V characteristics (see Fig. 5b–e).

Also, the carrier concentration of ⁓ 9.1 × 10^17^ cm^−3^, 2.3 × 10^19^ cm^−3^, and 3.6 × 10^18^ cm^−3^, for the N-500, N-550, and N-570 samples, respectively, was determined from the best fit of the experimental dark I–V data (Fig. 6b–d) using the following exponential fitting Eqs. (2) and (3) of the tunneling mechanism in the forward bias (Padovani-Stratton fit)^57^,

2 $$I=A \exp \left\{\frac{\mathrm{V}}{{E}_{00}\mathrm{coth}(\frac{{E}_{00}}{kT})}\right\}$$

3 $${E}_{00}= \frac{qh}{ 4\pi }\sqrt{\frac{{N}_{d}}{{m}^{*}\varepsilon}}$$

where A is the reverse saturation current, the exponential term in Eq. (2) is known as the tunneling parameter, E_00_ is the Padovani-Stratton parameter, q is the electronic charge, h is the Planck’s constant, m* is the electron effective mass, and ε is the semiconductor permittivity. The Padovani-Stratton parameter (E_00_) for N-500, N-550, and N-570 was found to be 20 meV, 100 meV, and 40 meV, respectively. The value of the carrier concentration is closer to the values extracted from UPS spectra and COMSOL (Fig. 6e). A decrease in carrier concentration with an increase in GaTe cell temperature is consistent with the variation of the carrier concentration with Te flux reported^59, 60^ in GaAs thin films, where carrier concentration peaks at 2–3 × 10^19^ cm^−3^ and reduces with further increase in dopant incorporation.

In NWs, Te dopant incorporation occurs predominantly through the Ga catalyst at the liquid–solid growth front interface via the VLS mechanism^16, 43^. Devkota et al.^43^ carried out a detailed morphological study on our samples reported in this work. A decrease in the aspect ratio of the NWs was observed with an increase in GaTe cell temperature from 500 to 570 °C. This is reproduced in the supplementary information (Figure S1(i)). With Te being a surfactant, the accumulated Te on the surface of the NW enhances the adatom adsorption rate, thereby decreasing the adatom diffusion length, resulting in increased diameter and decreased length of the NWs^61, 62^. It is to be noted that different BE shifts observed in As and Ga core level XPS spectra (disregarding Sb due to its low atomic percentage) and also, the difference in core level and valence band BE shifts show a complex dopant distribution in Te-doped NWs. Also, Bennett et al.^63^ observed surface segregation of Te in the MBE grown Te-doped GaAs thin films using a GaTe captive doping source at a high growth temperature of 600 °C by SIMS technique. At a higher GaTe cell temperature of 570 °C used in our work, a decrease in carrier concentration is observed. The Te induced defects have often been cited^59^ to be the cause for the reduction in carrier concentration at higher doping incorporation in thin films. However, the high surface to volume ratio in the one-dimensional NWs, the observed decrease in NW aspect ratio, the combination of both the high growth temperature of 590 °C along with high Te flux at a higher GaTe cell temperature of 570 °C, strongly suggests Te segregation is more likely the cause for the carrier concentration decrease. The doping inhomogeneity arising from segregation cannot be ruled out. It is to be pointed out that the inhomogeneity can potentially be suppressed^16^ using a higher V/III ratio for the NW growth.

**Figure 7:**
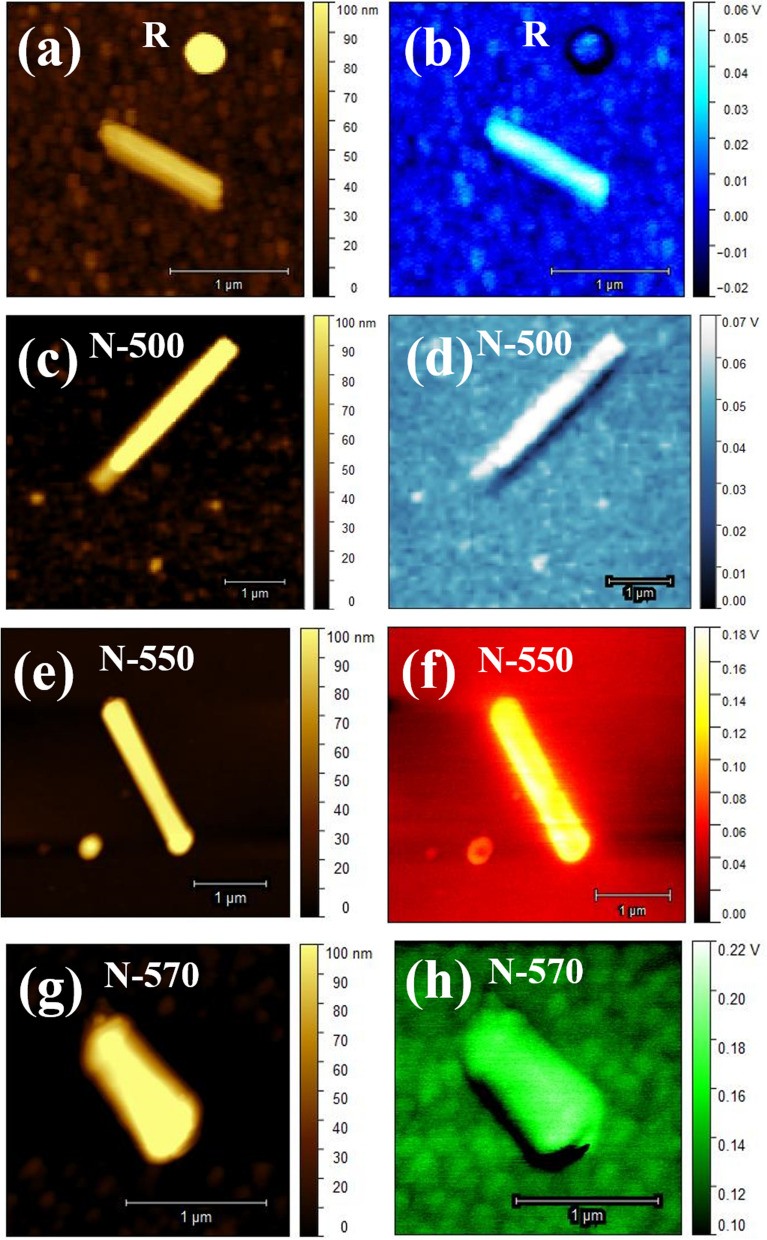
(**a**,**c**,**e**,**g**) Topography and (**b**,**d**,**f**,**h**) corresponding surface potential map of R, N-500, N-550, and N-570 sample, respectively, using SKPM.

**Figure 8:**
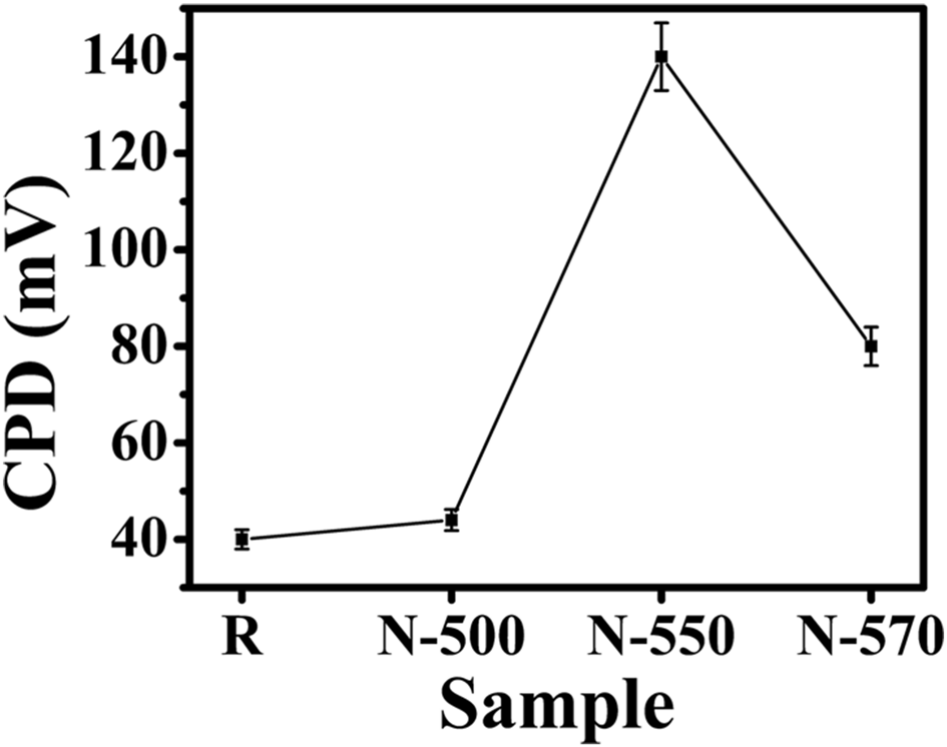
CPD between the NW sample and Au surface.

Finally, the SKPM technique has been used to obtain direct evidence of Te incorporation in N-500, N-550, and N-570 samples. For analyzing the change in contact potential difference (CPD) between the NW and Au deposited substrate, surface potential mapping using SKPM was carried out. Figure 7 shows the topography (Fig. 7a,c,e,g) and corresponding surface potential map (Fig. 7b,d,f,h) of R, N-500, N-550, and N-570 sample, respectively. Topography maps of R and N SNW samples (Fig. 7a,c,e,g) reveal the average surface roughness of ~ 2.2 nm and ~ 4.9 nm, respectively. An increase in surface roughness in the N samples is due to the surfactant nature of Te^61^ and can be reckoned to be indicative of Te incorporation. The following Eq. (4) shows the CPD between the NW and Au surface (CPD_(NW-Au)_)^64, 65^.

4 $${\text{CPD}}_{{({\text{NW}} - {\text{Au}})}} = {\text{ W}}_{{{\text{Au}}}} {-}{\text{ W}}_{{{\text{NW}}}}$$

where W_Au_ and W_NW_ are the work functions of Au and NW samples, respectively. An increased CPD between the NW and Au surface can be observed in the N samples (Fig. 8), reflecting reduced work function and hence is direct evidence of Te incorporation^66^. Figure 8 reveals the highest CPD of ~ 140 mV between the N-550 sample and the Au surface. This result confirms high Te incorporation in the N-550 sample, which is consistent with the UPS measurements, where the reduction in work function is observed in the N-550 sample. However, as the measurement is carried out at room temperature, the biased voltage, probe tips, and also the presence of absorbent and surface states in the nanoscale regime have a significant effect on the surface potential measurement. Hence, quantitative analysis was not performed.

From the above measurements, it can be observed that the N-550 sample is representative of a heavily doped n-type sample with Te. Also, the increase in conductivity and CPD from C-AFM/SKPM measurements attest to the Te incorporation in the N samples. From XPS, incorporation of Te dopant was confirmed, while the carrier concentration of the N-550 sample obtained from UPS and C-AFM (Padovani – Stratton fit) was 2.8 × 10^19^ cm^−3^ and 2.3 × 10^19^ cm^−3^, respectively. A carrier concentration of 4 × 10^19^ cm^−3^ was extracted from an independent simulation of the curve fitting of the I–V characteristics of ensemble doped NWs using two probe I–V reported elsewhere^43^.Though there is some variation in the carrier concentration, these values can be viewed as excellent agreement as these data are from the single nanowire, ensemble NWs and also probing depth for the data collection are also quite different. Thus, the remarkable agreement between the surface composition, energy bands, carrier, and dopant concentration using the surface analytical techniques with the corresponding values extracted from the electrical measurements shows that the dopant incorporation is homogeneous throughout the NW along with good uniformity amongst the NWs. In such a case, the surface analysis technique can provide reliable data that is representative of the entire NW. Thus, the combination of XPS/UPS and C-AFM/SKPM tools have been demonstrated to be a simple, direct, promising method for evaluating the dopant concentration in a homogeneously doped NW.

## Methods

The intrinsic and Te-doped NWs were grown in an EPI 930 solid source MBE system with valved As_4_ and Sb_2_ as the group V constituent cracker sources. The self-catalyzed GaAs stem/GaAsSb core NWs were grown on a chemically cleaned (Piranha/HF) p-type Si (111) substrate at 620 °C/590 °C^43^. The intrinsic GaAsSb NWs were grown with a constant group III flux of 8 × 10^–6^ mbar, and group V flux of 8 × 10^–7^ mbar and GaTe cell temperatures of 500 °C, 550 °C, and 570 °C were used for doped NWs.

Morphological characterization of intrinsic and doped NWs was carried out using Carl-Zeiss Auriga-BU FIB FESEM. XPS/UPS measurements were performed with a Thermo ESCALAB 250 Xi using monochromated Al Kα X-rays source (hν = 1486.6 eV) and He-I photon source (21.21 eV), respectively. The base chamber pressure was kept in the 10^–9^ mbar range at room temperature, and the diameter of the analyzed area was about 300 µm. XPS survey scans of both R and N samples were acquired across binding energy (BE) range from 0 to 1370 eV with a pass energy of 200 eV and an energy step-size of 1 eV, while the core-level high-resolution scans were performed with an energy step-size of 0.1 eV. The XPS energy scale was calibrated with the binding energy of the photoelectron peak of C 1s at 284.8 eV from the gold substrate sample. The elements scanned from R, N-500, N-550, and N-570 samples, are gallium (Ga 3d), arsenic (As 3d), and antimony (Sb 3d) core-levels. The peak fitting was done by the Avantage analysis software program using a G-L product function with a mix ratio (L/G) of 30% and a smart-type background, which is Shirley BG with the additional constraint that the BG should not be greater than the actual intensity at any point in the selected region. The work function and valence band energy of R and N samples were obtained from UPS. The photoemission spectra were collected at 2 eV pass energy under a stable reverse bias of 4 V to eliminate an instrumental cutoff in the lens system of the analyzer at low kinetic energy.

The electrical characteristics have been investigated with C-AFM MFP-3D Origin (Asylum) in ambient conditions, operating in the contact mode. In conjunction with the topography of the sample, corresponding current mapping under a specific bias of 600 mV and a scan rate of 0.2 Hz is recorded. An I–V measurement was carried out on a SNW by positioning the AFM probe onto the nanowire tip and the other contact at the NW substrate, and varying voltages between − 1.5 and 1.5 V under dark and laser illumination of 860 nm wavelength. As the probe tip is much smaller than the diameter of NWs (order of 100 nm), SNW I–V characteristics were performed accurately with high spatial resolution^30^. Charge mobility of R and N samples were determined using COMSOL Multiphysics fitting of I–V. SKPM scanning of the R and N samples was carried out using lift mode, which uses two separate passes per scan line. The sample topography is measured in the first pass, while the surface potential is measured in the second pass. Ti/Ir conductive tips (Asylum) with a typical radius of curvature of 25 nm at a resonant frequency of 75 kHz were used for both C-AFM and SKPM techniques.

## Conclusions

XPS/UPS and C-AFM/SKPM were successfully used to ascertain the Te incorporation and electron concentration in Te-doped GaAsSb NWs samples grown by varying GaTe cell temperatures. Te-dopant incorporation in GaAsSb NWs was determined from the XPS spectroscopic shift of the binding energies of the core constituent elements towards higher energy. The lowering of the work function manifested by a positive shift in the binding energy of the UPS spectra of Te-doped GaAsSb NWs grown at the GaTe cell temperature of 550 °C revealed the sample to be a heavily n-doped sample with a carrier concentration of 2.8 × 10^19^ cm^−3^. This value is in excellent agreement with the carrier concentration of 2.3 × 10^19^ cm^−3^ obtained from dark I–V measurements on SNW by C-AFM. C-AFM showed enhanced photoresponse in doped SNW samples compared to intrinsic SNW corroborating Te incorporation and an increase in carrier concentration results from XPS/UPS. The increase in surface potential observed in doped NWs in SKPM characterization also provided strong evidence of Te incorporation. The different binding energy shifts in As and Ga core levels and between core levels and valence band suggest a complex dopant distribution in the NWs. Finally, the decrease of carrier concentration at higher GaTe cell temperature of 570 °C along with high growth temperature attributed to the Te segregation in the NWs.

## Supplementary Information

Supplementary Information.
