# Supplementary material for: A study of dopant incorporation in Te-doped GaAsSb nanowires using a combination of XPS/UPS, and C-AFM/SKPM
Source: Sci Rep. 2021 Apr 15;11:8329. doi: 10.1038/s41598-021-87825-4 (PMC8050051; doi:10.1038/s41598-021-87825-4)
Supplement: Supplementary file 1 — Supplementary Information. [file 41598_2021_87825_MOESM1_ESM.pdf]

Supplementary material

A Study of Dopant Incorporation in Te - doped GaAsSb Nanowires using a Combination of XPS/UPS, and C-AFM/SKPM.

Priyanka Ramaswamy [1], Shisir Devkota [2], Rabin Pokharel [2], Surya Nalamati [1], Fred Stevie [3], Keith Jones [4], Lew Reynolds [5], and Shanthi Iyer [2,*]

[1] Department of Electrical and Computer Engineering, North Carolina A&T State University, Greensboro, North Carolina 27401, USA.
[2] Nanoengineering, Joint School of Nanoscience and Nanoengineering, North Carolina A&T State University, Greensboro, North Carolina 27401, USA.
[3] Analytical Instrumentation Facility, North Carolina State University, Raleigh, NC 27695, USA.
[4] Asylum Research, an Oxford Instruments Company, 6310 Hollister Ave. Santa Barbara, CA 93117, USA.
[5] Department of Materials Science and Engineering, North Carolina State University, Raleigh, NC 27695, USA.
* Correspondence: iyer@ncat.edu

Scanning electron microscope images of NWs of low magnification (Figures S1 (a, c, e, and g)), high magnification (Figures S1(b, d, f, and h)), and variation of the aspect ratio of NW samples with GaTe cell temperature from Devkota et al. from Nanotechnology, 31, 505203 (2020) are shown below.

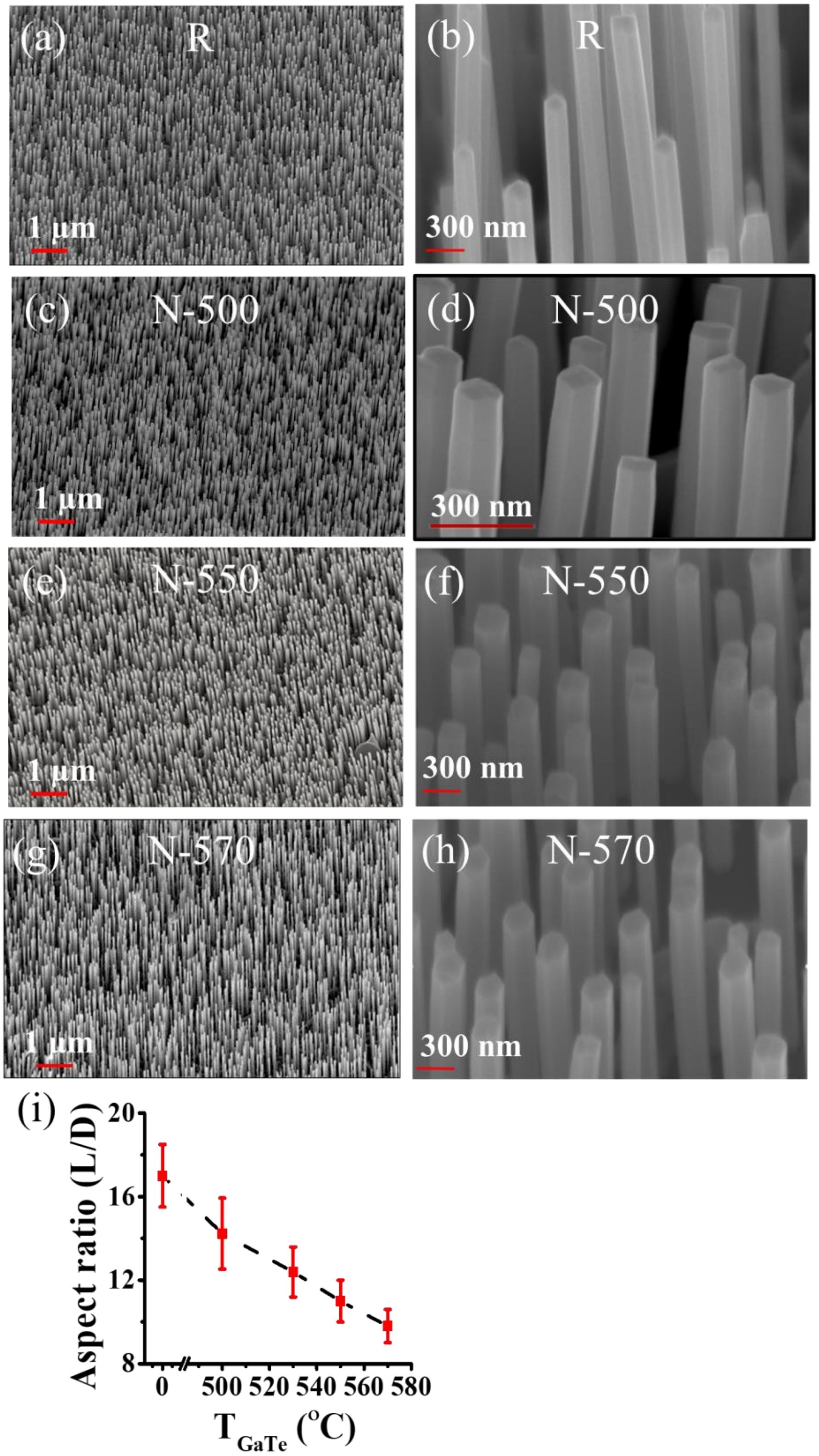

Figure S**1.** (a, c, e, g) Low magnification and (b, d, f, h) high magnification SEM images of R, N-500, N-550, and N-570 samples, respectively, and (i) variation of the aspect ratio of NW samples with GaTe cell temperature.

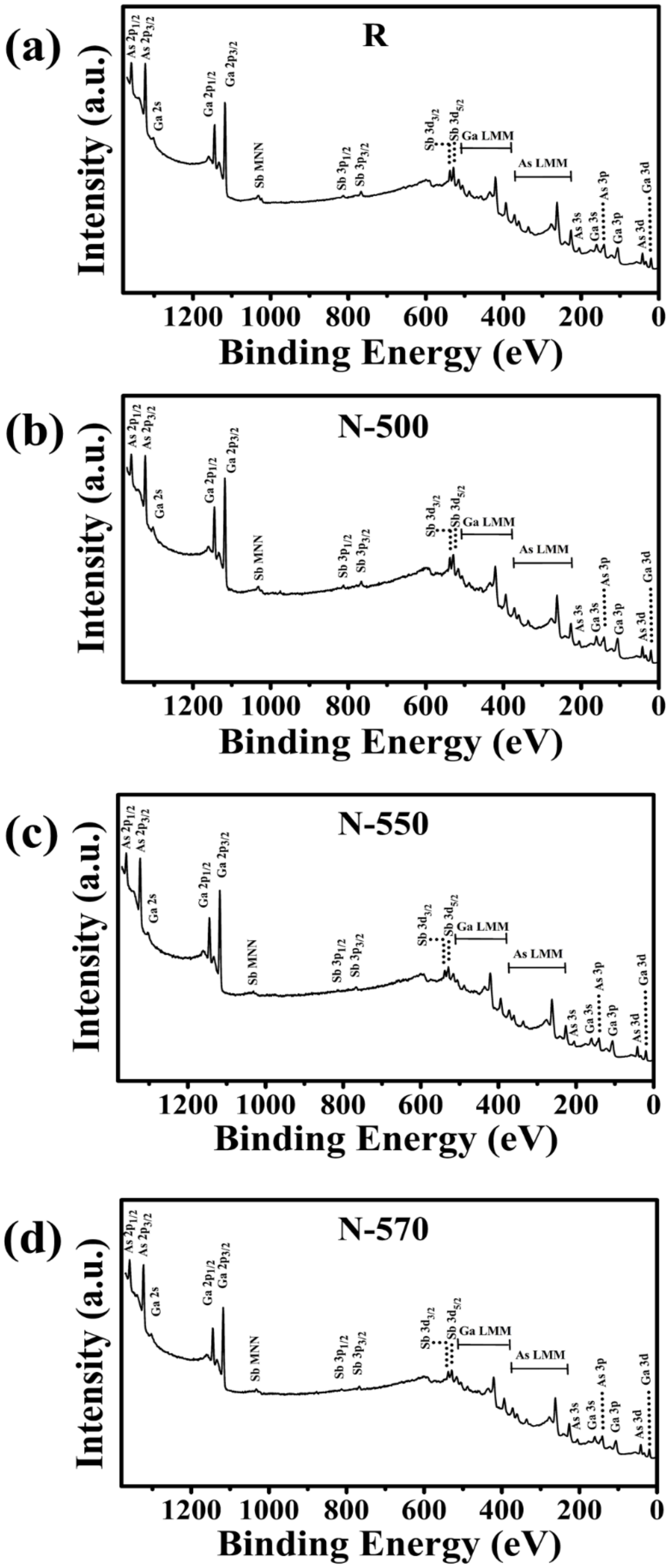

Figure S**2.** XPS spectra of (a) R, (b) N-500, (c) N-550, and (d) N-570 samples.

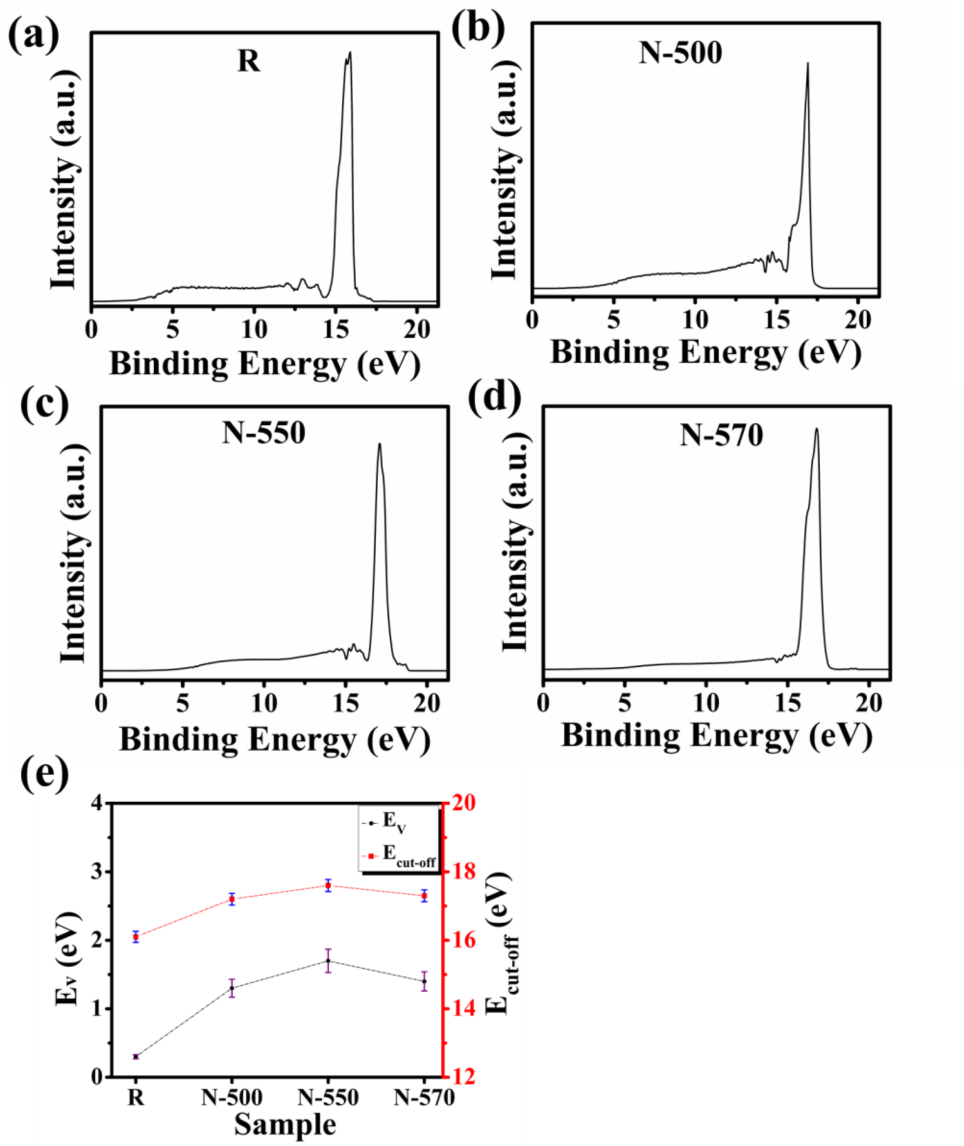

Figure S3. UPS spectra of (a) R, (b) N-500, (c) N-550, and (d) N-570 samples and (e) E_v and $E_{cut\text{-}off}$ of R, N-500, N-550, and N-570 samples using UPS.

From the comparison of the valence band lineshape in all materials (figures S3 (a-d)), it seems that at least the filled density of states does not change. Assuming similar invariance in conduction band's empty density of states in these NWs, the following equations S1 and S2 are used in the calculation of carrier concentration from UPS using equation 1.

$$\eta_F = \frac{E_F - E_C}{kT} \qquad \text{(S1)}$$

$$F_{1/2}(\eta_F) = \int_0^{\infty} \frac{\eta^{1/2}\, d\eta}{1+\exp(\eta-\eta_F)} \qquad \text{(S2)}$$

Where E_F and E_C represent the Fermi energy and conduction band energy, respectively. kT is the product of Boltzmann's constant and temperature, and $F_{1/2}(\eta_F)$ is the Fermi-Dirac integral.
